# Study on dynamic resilient modulus prediction model of subgrade fine-grained soil based on physical property parameters

**DOI:** 10.1038/s41598-025-02070-3

**Published:** 2025-05-21

**Authors:** Fang Guo, Wei Gu

**Affiliations:** 1 Hunan Communication Polytechnic, Changsha, 410132 Hunan China; 2 Hunan Communications Research Institue Co., Ltd., Changsha, 410015 Hunan China

**Keywords:** Subgrade fine-grained soil, Dynamic resilient modulus, Prediction model, Artificial neural network, Physical property parameters, Engineering, Civil engineering

## Abstract

This study took a representative subgrade fine-grained soil of a highway as the research object, prepared specimens with different water content and compaction degrees, and investigated the effects of water content, dry density, plasticity index, dynamic bias stress and circumferential pressure on the dynamic resilient modulus *M*_r_ by dynamic triaxial test. Subsequently, using the data from this study and previous research, a correlation analysis was conducted on the factors affecting the resilient modulus, identifying the factors that contribute significantly to the resilient modulus. The relationship between *k*_*i*_ parameters and key physical property parameters was acquired by utilizing artificial neural network and genetic algorithm. Finally, a prediction model considering physical property parameters was established, and the prediction results were compared with those of the general model in standard. The study results show that *M*_r_ decreases nonlinearly with the decrease in dynamic deviator stress and moisture content and increases with the increase in confining pressure and compaction degree, and *M*_r_ is uniformly and inversely related to *I*_p_. Acoording to Spearman correlation coefficient values between various input features and the resilient modulus, *M*_r_ has the strongest correlation with the moisture content, dry density, plasticity index and fine particle content. Compared with the standard universal model, the model proposed in this paper has an average prediction error of 5.74%, and the maximum prediction error is not more than 15%, which means the prediction effect is improved. Therefore, for a specific highway subgrade construction project, it is valuable to carry out targeted dynamic resilient modulus in advance to establish a prediction model based on physical properties indicators.

## Introduction

The dynamic resilient modulus *M*_r_, as the main mechanical index to measure the deformation resistance of compacted soil under traffic loading, is defined as the ratio of cyclic bias stress to resilient strain^1^. Its magnitude directly influences the prediction of fatigue cracking of pavements and the design of thickness. However, subgrade compacted soil is a three-phase body composed of solid particles, water, and gas, and its mechanical properties are much more complex than those of general metallic and nonmetallic materials.

Numerous scholars have investigated the factors influencing the resilient modulus (*M*_r_), including deviator stress^2–4^, fine content^5^, degree of compaction^6^, moisture content^7,8^, freeze–thaw cycles^9^ and loading frequency^10^. With the evolution of roadbed and pavement design philosophies, an increasing number of standards have begun to adopt the dynamic resilient modulus, which better reflects the impact of traffic loads on the roadbed, to characterize the mechanical properties of the roadbed. Examples include the AASHTO 2004 Pavement Design Guide and the Mechanistic-Empirical Pavement Design Guide (MEPDG)^11^, as well as Chinese"Design specification for highway subgrade" (JTG D30-2015)^12^. The prerequisite for determining the dynamic resilient modulus of the roadbed is the estimation of the dynamic resilient modulus of the subgrade soil. Over the years, many scholars, both domestically and internationally, have established various predictive models for the dynamic resilient modulus based on in-depth analyses of its primary influencing factors, such as the stress state^13,14^, moisture state^15,16^, and physical property indices^17,18^ of the subgrade soil. Due to differences in the main factors considered by each model and regional geological environments, the applicability of these models varies. Overall, Zhang et al.^19^ categorized resilient modulus models into three types through extensive literature analysis: Type A, models that use stress as a variable; Type B, models that couple matric suction with stress variables; and Type C, models that treat matric suction as an independent stress variable. Although empirical models are relatively simple in form and can provide accurate predictions on specific datasets, they often require numerous parameters and have poor generalization capabilities, especially when addressing multifactorial problems, necessitating extensive experimental modeling and consuming significant resources, thereby limiting their applicability.

In recent years, intelligent algorithms, particularly artificial neural networks, have been applied to the prediction of subgrade soil performance. These data-driven methods are not confined to fixed equation forms, offering greater flexibility. Xu^20^ employed the XGBoost algorithm to predict the resilient modulus of the subgrade, and considering the numerous hyperparameters in XGBoost, combined it with Bayesian Optimization (BO) for hyperparameter tuning. Tan^21^ established a relationship model between the dynamic resilient modulus of the subgrade and moisture content, degree of compaction, fine content, and plasticity index using an artificial neural network algorithm, with results showing that the deviation between the estimated and measured dynamic resilient modulus was around 5%. Heidaripanah et al.^22^ used the Support Vector Machine (SVM) method to establish a quantitative relationship between the dynamic resilient modulus and confining pressure, deviator stress, moisture content, and dry unit weight, and explored the impact of different kernel functions on prediction results. Kardani et al.^23^ employed four machine learning algorithms-Gradient Boosting, Decision Trees, K-Nearest Neighbors, and Random Forests-to establish correlations between the resilient modulus and dry density, weighted plasticity index, deviator stress, confining pressure, freeze–thaw cycles, and moisture content, achieving accurate estimation of the subgrade resilient modulus. However, traditional machine learning methods are often considered "black-box models," with poor interpretability and non-intuitive predictive models, leading some engineers to question the reliability of machine learning predictions^24^. Additionally, the inclusion of parameters with low correlation to the resilient modulus in these models can increase model complexity and reduce generalization capabilities.

To leverage the advantages of intelligent algorithms in addressing multifactorial problems while maintaining the usability and intuitiveness of traditional empirical models, this study combines self-conducted experimental data with previous research data to explore the variation patterns of the resilient modulus and analyze the significance of various influencing factors. Building on the MEPDG recommended model, a hybrid approach—combining artificial neural networks with genetic algorithms—was employed for global parameter search, establishing a predictive model for the dynamic resilient modulus that considers physical property indices. The predictive results were then compared with those from commonly used empirical models and AI algorithm models.

## Test procedure of dynamic resilient modulus of subgrade soil

**Fig. 1:**
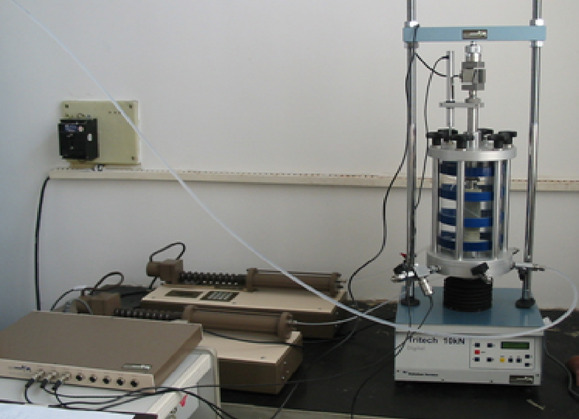
GDS dynamic triaxial test system.

The study area is located in a subtropical monsoon humid climate zone, characterized by rugged terrain and complex, diverse geomorphological types, with a predominance of mountainous regions and fewer hilly plains. The route features numerous high-fill embankments, resulting in a significant demand for subgrade filling materials. In order to reduce the cost of purchasing and transporting soil from external sources, a large number of fine-grained soils distributed along the line was used. To study the dynamic resilient modulus properties of the main road construction fill, corresponding tests for determining *M*_r_ were carried out referring to Chinese “Design specification for highway subgrade”^12^ (JTG D30-2015). The experimental apparatus is the GDS dynamic triaxial test system (Fig. 1). First, equal confining pressure *σ*_3_ was applied to the specimen from all directions, and then cyclic deviatoric stress *σ*_d_ (half-sine waveform) was applied in the axial direction. Each specimen is firstly loaded 1000 times to ensure that the permanent deformation is basically stable, and then loaded 100 times, and the last 5 times of the resilient strain (*ε*_r_) are taken as the basis for the calculation of the resilient modulus. The resilient modulus is determined by the following equation:

1 $$M_{{\text{r}}} = \sigma_{{\text{d}}}^{{}} /\varepsilon_{{\text{r}}}$$

where *σ*_d_—cyclic deviatoric stress(kPa);*ε*_r_—resilient strain.

The loading process is not drained so that the specimen can maintain a constant water content. The loading stresses were selected to be representative of the stress state of the subgrade soil, where 15 kPa, 30 kPa, 45 kPa and 60 kPa were used for the confining pressure *σ*_3_, and 30 kPa, 55 kPa, 75 kPa and 105 kPa were used for the cyclic deviatoric stress *σ*_d_. According to Chinese “Design specification for highway subgrade”^12^ (JTG D30-2015), the loading frequency for testing *M*_r_ is set to 10Hz.

Six sets of representative specimens were taken from the main soil extraction sites along the route. In accordance with the current “Test Methods of Soils for Highway Engineering” (JTG3430-2020)^25^, the basic physical property indexes such as natural water content, maximum dry density, bounding water content, and granular gradation were obtained from indoor tests as shown in Table 1. The *w*_L_ are all less than 50%, which belongs to low liquid limit soil. Soil particles with a grain size of 0.075–2 mm are classified as sand particles, while particles smaller than 0.075 mm are classified as fine-grained soil particles, including silt particles and clay particles.

**Table 1:** Basic physical properties of tested soil.

Soil types	Natural water content *w*/%	Max. dry density *ρ*_dmax_/(g.cm^−3^)	Optimum water content *w*_opt_/%	Liquid limit *w*_L_/%	Plastic limit *w*_P_ /%	Plasticity index *I*_p_/%	USCS	Particle Size distribution /%	
								< 0.075 mm	> 2 mm
No. I	24.8	1.82	19.6	38.1	25.2	12.9	ML	53.4	1.2
No. II	25.6	1.75	20.2	46.3	20.6	25.7	CL	69.2	4.5
No. III	24.1	1.78	19.8	40.1	25.1	15.0	CL	60.7	3.2
No. IV	23.8	1.85	20.1	39.7	24.0	18.7	CL	59.1	1.4
No. V	25.8	1.80	18.3	43.2	21.2	22.0	CL	64.2	2.3
No. VI	22.9	1.91	20.5	35.7	23.8	11.9	CL	55.1	1.2

**Fig. 2:**
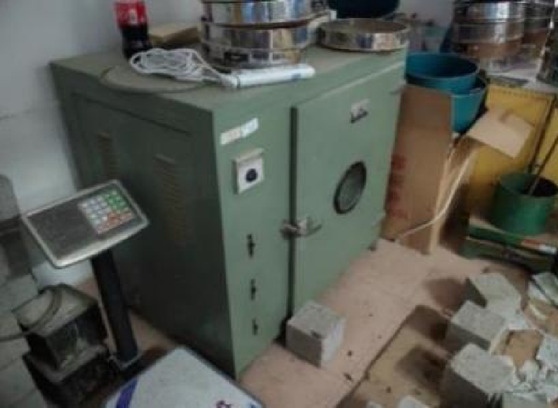
Drying for soil in natural condition.

The collected natural soils were dried in an oven (Fig. 2). Then, the dried soil was compacted in layers in the mold to form a cylindrical specimen with dimensions of 50 mm in diameter and 100 mm in height. A rubber membrane was fitted over the specimen to ensure airtight sealing. The specimen was then placed on pre-soaked moist porous permeable stones and a bottom platen, with pre-soaked permeable stones and a top platen added above. The assembled specimen was positioned at the center of the triaxial chamber base, ensuring alignment between the specimen center and the loading frame center.

The specimens were prepared under three different water contents (*w* = *w*_opt,_ *w*_opt_ + 4%, and *w*_sat_) and three compaction degrees (*K* = 93%, 94%, and 96%), and the numbering of the specimens was shaped like I-93-1 (I stands for the soil No. I, 93 stands for the compaction degree of 93%, and -1, -2, and -3 stand for the water contents of *w*_opt_, *w*_opt_ + 4%, *w*_sat_, respectively). The compaction degree *K* represents the ratio of the specimen’s dry density to its maximum dry density, serving as an indicator of the specimen’s compaction state. To determine the saturated water content (*w*_sat_), the specimen shall first be saturated through water immersion. When the pore water pressure coefficient *B* exceeds 95%, the soil is considered fully saturated. Subsequently, samples are taken to measure the water content, which is recorded as *w*_sat_.

## Analysis of test results

The following analysis primarily takes Soil I as an example to investigate the factors influencing the dynamic resilient modulus (*M*_r_) of subgrade soil.

### Stress state effects

**Fig. 3:**
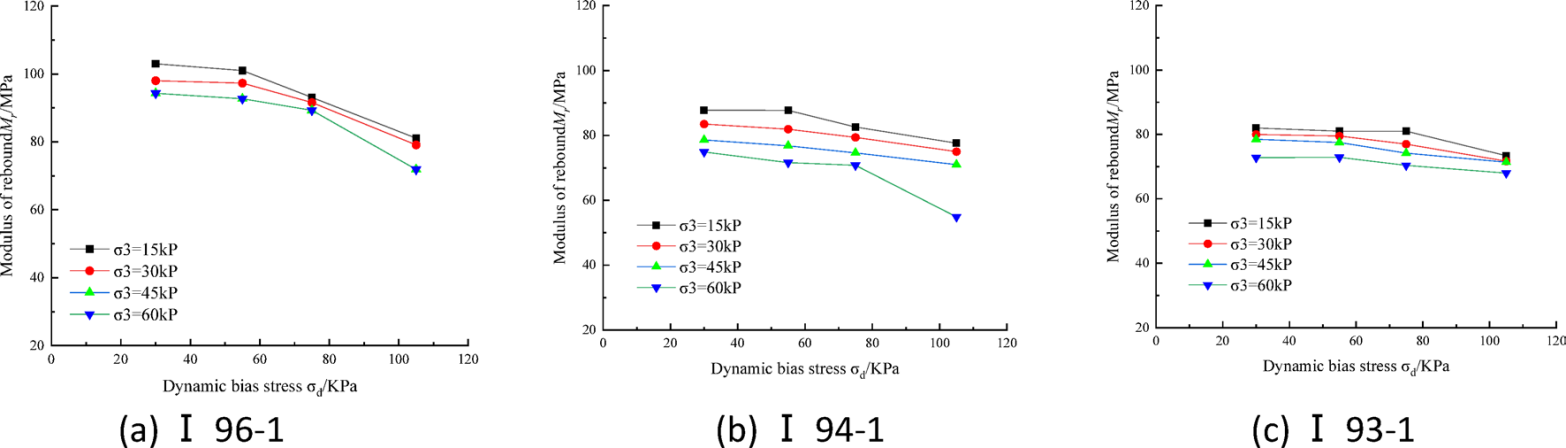
Dynamic resilient modulus-stress state relation of soil I

Figure 3 shows the variation curves of *M*_r_ with stress state for soil specimen No. I at optimum moisture content and different compaction degrees, *M*_r_ decreases nonlinearly with the increase of dynamic bias stress, which agrees with the trends observed in literature^14,17^. Under the same circumferential pressure, the decrease range is from 5.6 to 26.7%. *M*_r_ rises with increasing perimeter pressure and the increase ranges from 8.7 to 36.8% at the same dynamic bias stress. It can be seen that both dynamic bias stress and circumferential pressure have a greater effect on the subgrade fine-grained soil *M*_r_. The shear displacement caused by the increase of dynamic bias stress has a destructive effect on the fine-grained soil structure, while the increase of circumferential pressure improves the occlusion and embedding effect between the fine particles, which in turn improves the overall stiffness of the specimen.

### Effect of compaction

**Fig. 4:**
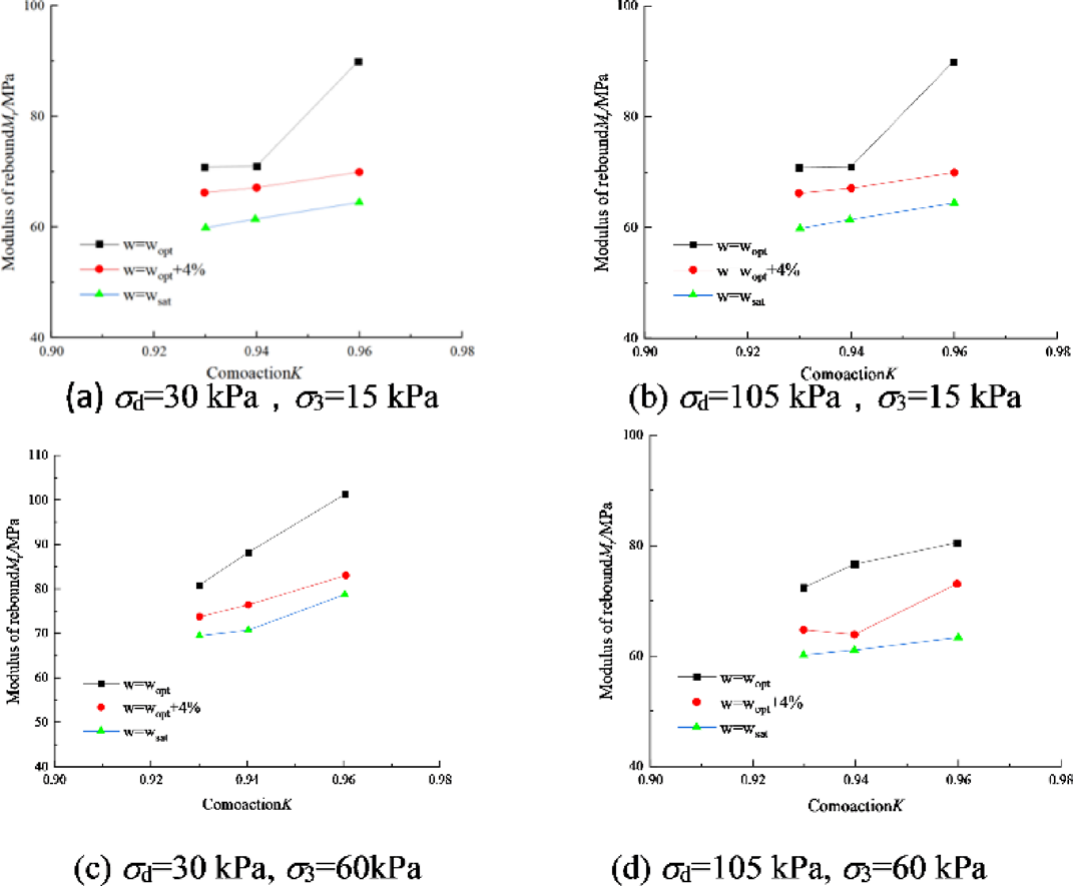
Dynamic resilient modulus-compaction degree relation of soil I

Figure 4 shows the variation curves of *M*_r_ for soil specimen No. I with compaction degree *K* under different stress states. With the increase of compaction degree, the soil particles are arranged more tightly. So *M*_r_ is improved, among which the increase of compaction degree has the most obvious effect on the enhancement of *M*_r_ under the optimal water content, and *M*_r_ basically rises linearly with the increase of compaction degree under the conditions of both *w*_opt_ + 4% and *w*_sat_.

### Effect of moisture content

**Fig. 5:**
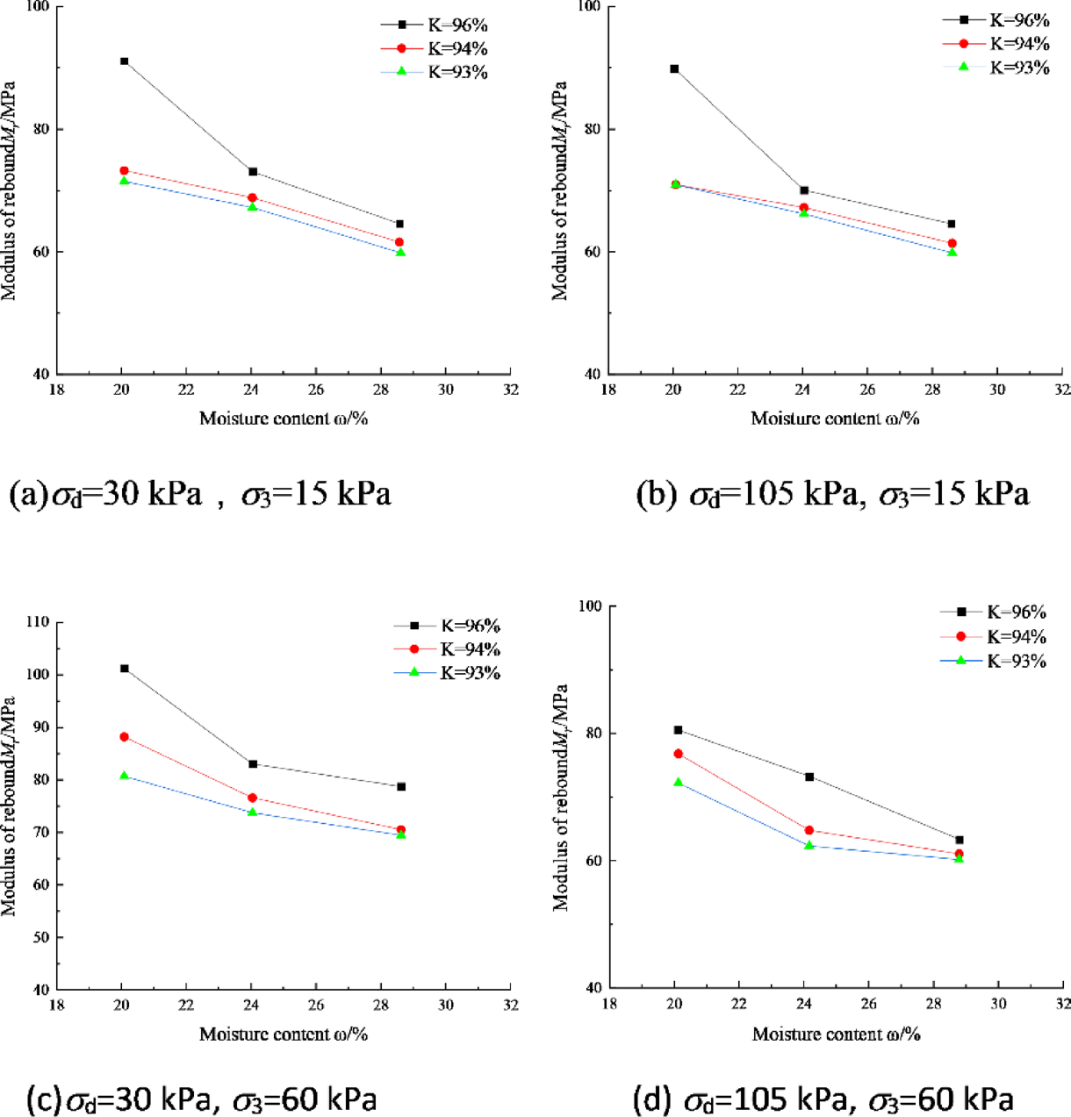
Dynamic resilient modulus-moisture content relation of soil I

Figure 5 shows the variation curves of *M*_r_ with water content *w* for soil specimen No. I under different stress states. It can be seen that *M*_r_ decreases nonlinearly as the water content increases, which is consistent with the findings reported in literature^19^. When the water content increases from 19.6 to 28.7%, the *M*_r_ of No. I soil decreases by 19% on average, i.e., for every 1% increase in water content, *M*_r_ decreases by 2.1% on average. Moreover, the higher the compaction degree is, the more sensitive *M*_r_ is to the change in water content. The cohesion of fine-grained soil depends on factors such as inter-particle gravitational force, matrix suction, cementation, etc. When the water content reaches *w*_opt_ and then continues to increase, the water will be mainly in the form of pore water endowed in the soil. On one hand, this leads to the thickening of the weakly bonded water film, the increase in the spacing between particles, and the decrease in inter-particle gravitational force. On the other hand, it makes the degree of saturation increase, and the matrix suction decreases rapidly. Therefore, the dynamic resilient modulus of compacted fine-grained soil of the subgrade decreases significantly with the increase of water content.

### Effect of plasticity index

**Fig. 6:**
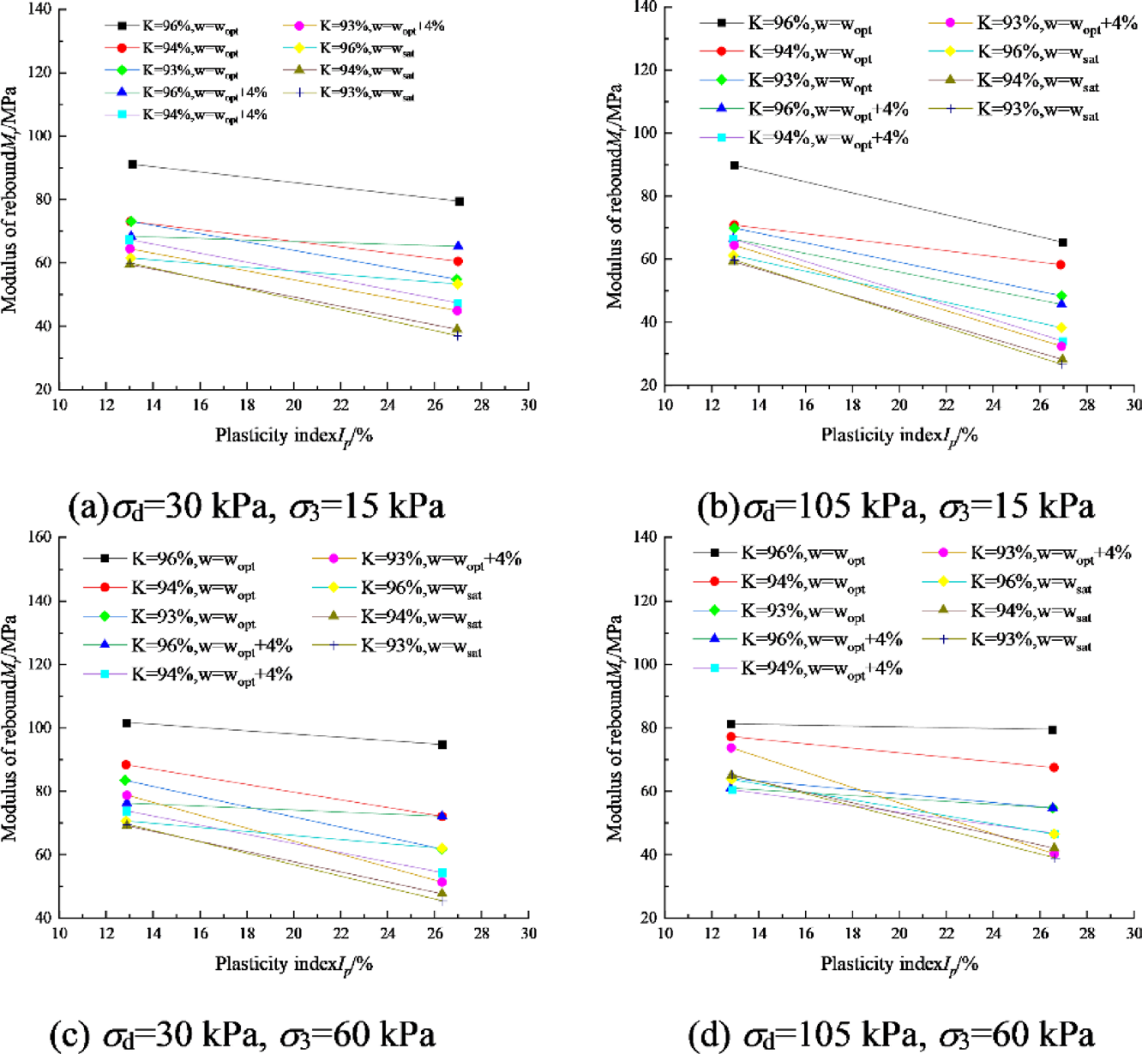
Dynamic resilient modulus-plasitc index relation of soil I and soil II

Figure 6 shows the variation curves of *M*_r_ with plasticity index *I*_p_ for two kinds of soil specimens of I and II, and it can be seen that *M*_r_ is uniformly inversely proportional to *I*_p_, and the engineering should avoid using fine-grained soil with too high a plasticity index for subgrade filling as far as possible. Soils with higher plasticity index (*I*_p_) generally contain significant amounts of highly water-absorbent clay minerals such as montmorillonite. Upon water absorption, these minerals develop thick hydration films that reduce interparticle effective stress and consequently decrease the soil’s resistance to elastic deformation. In contrast, soils with lower *I*_p_ exhibit thinner water films, where enhanced interparticle friction and interlocking effects result in greater resistance to elastic deformation and higher resilient modulus.

## Parameter correlation analysis

In reality, the magnitude of the resilient modulus of a roadbed is governed by multiple interacting factors, forming a complex, dynamic nonlinear system. To ensure the generalization capability of the data, this study collected *M*_r_ test data from different types of compacted fine-grained subgrade soils, comprising more than 1500 datasets, as shown in Table 2. The dataset is partitioned into training and test sets in an 8:2 ratio. These datasets include multiple feature components such as geotechnical mechanics, soil properties, environmental parameters, and *M*_r_ itself. Key parameters include the plasticity index (*I*_p_), dry density (*γ*_d_), moisture content (*w*), among others.

**Table 2:** Physical properties of different soil specimens.

No.	*G*_s_	*w*_L_/%	*w*_P_%	*I*_p/_%	*W*_opt_/%	*P*_dmax_/(g ·cm⁻^3^)	Sand/%	Clay/%	Data source
#1	2.72	35.5	16.5	19.0	15.5	1.884	28.0	30.0	Literature^26^
#2	2.68	19.6	13.6	6.0	13.5	1.954	3.0	16.0	Literature^27^
#3	2.75	48.0	22.0	26.0	23.0	1.649	20.0	32.0	
#4	2.71	31.0	21.0	10.0	20.3	1.664	15.0	25.0	
#5	2.74	32.5	18.5	14.0	18.2	2.002	10.0	20.0	
#6	2.69	25.0	13.0	12.0	12.2	1.806	31.0	19.0	
#7	2.67	31.0	24.0	7.0	17.0	1.735	35.0	13.0	Literature^28^
#8	2.65	38.0	21.0	17.0	16.0	1.765	19.0	18.0	
#9	2.71	44.0	18.0	26.0	22.0	1.612	4.0	35.0	
#10	2.66	88.0	35.0	53.0	35.0	1.255	2.0	84.0	
#11	2.72	27.8	19.8	8.0	14.2	1.847	43.7	16.3	Literature^29^
#12	3.69	30.8	18.4	12.3	16.5	1.842	31.2	13.8	
#13	2.69	42.0	18.0	24.0	22.0	1.612	8.9	27.3	Literature^30^
#14	2.66	26.0	17.0	9.0	16.0	1.806	36.2	14.5	
#15	2.66	26.0	17.0	9.0	16.0	1.806	36.2	14.5	
#16	2.69	28.0	17.0	11.0	13.5	1.827	11.9	5.7	
#17	2.75	85.0	33.0	52.0	27.5	1.469	3.6	75.2	
#18	2.64	27.5	16.5	11.0	13.6	1.980	14.0	10.0	Literature^31^
#19	2.72	70.8	35.2	35.6	18.5	1.720	23.9	42.6	Literature^32^

**Fig. 7:**
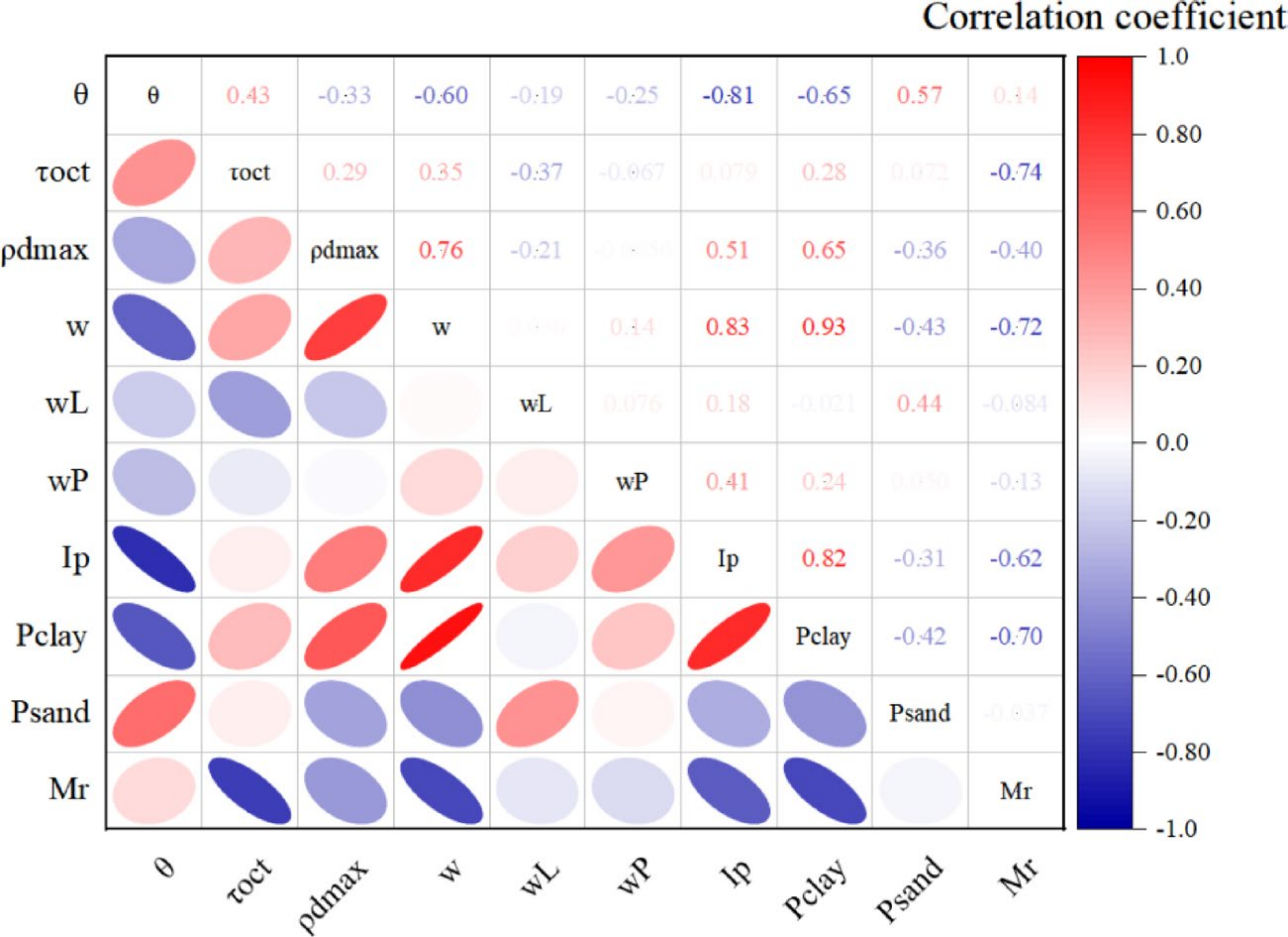
Nonlinear correlation among variables expressed by Spearman correlation coefficients.

The Spearman correlation coefficient is a non-parametric statistical method based on ranks, used to measure the correlation between two variables. It is suitable for data that does not meet the assumption of a normal distribution and can detect non-linear relationships. This method transforms the original data into their ranks and then calculates the correlation between the ranks to assess the relationship between the variables. Its value ranges from − 1 to 1, where − 1 indicates a perfect negative correlation, 0 indicates no correlation, and 1 indicates a perfect positive correlation. Compared to the Pearson correlation coefficient, the Spearman correlation coefficient is more robust to outliers, making it widely adopted in practical applications. Figure 7 displays the Spearman correlation coefficient values between various input features and the resilient modulus. It can be observed that, apart from the correlation coefficients for body stress and octahedral shear stress in MEPDG model, *M*_r_ has the strongest correlation with the moisture content *w* (%), dry density *ρ*_d_ (g/cm^3^), plasticity index *I*_p_ (%), and fine particle content *P*_0.075_ (%).

## Modeling of prognostications considering physical property indicators

### Parameter search method

According to the above analysis, both the perimeter pressure and the bias stress have a significant effect on the dynamic resilient modulus of the subgrade soil. Therefore, the dynamic resilient modulus prediction model should be a composite model that can consider both the effect of perimeter pressure and the effect of bias stress, and it should solve the problem of the absence of magnitude and indeterminate value. A representative composite model that considers this phenomenon is such as the model in AASHT02002^11^:

2 $$M_{{_{r} }} = k_{1} P_{a} \left( {\frac{\theta }{{P_{a} }}} \right)^{{k_{2} }} \left( {\frac{{\uptau _{oct} }}{{P_{a} }} + 1} \right)^{{k_{3} }}$$

where *θ* = *σ*_1_ + *σ*_2_ + *σ*_3_; $\tau_{{{\text{oct}}}} = \sqrt {\left( {\sigma_{1} - \sigma_{2} } \right)^{2} + \left( {\sigma_{1} - \sigma_{3} } \right)^{2} + \left( {\sigma_{2} - \sigma_{3} } \right)^{2} } /3$; *k*_*i*_—model parameter; *p*_a_—Reference Atmospheric Pressure (100 kPa).

**Fig. 8:**
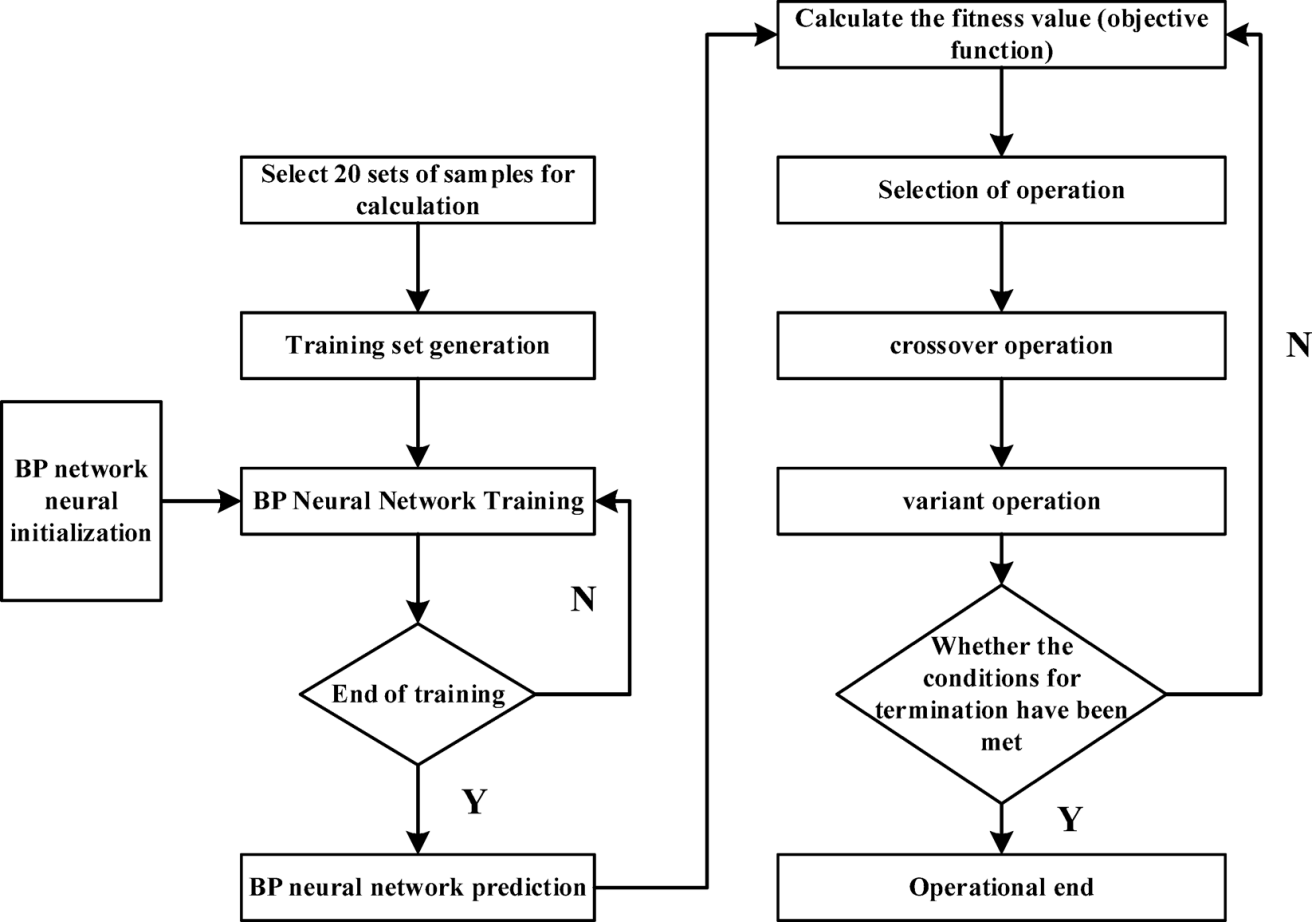
The process of establishing the relationship formula between *k*_*i*_ and physical index.

**Fig. 9:**
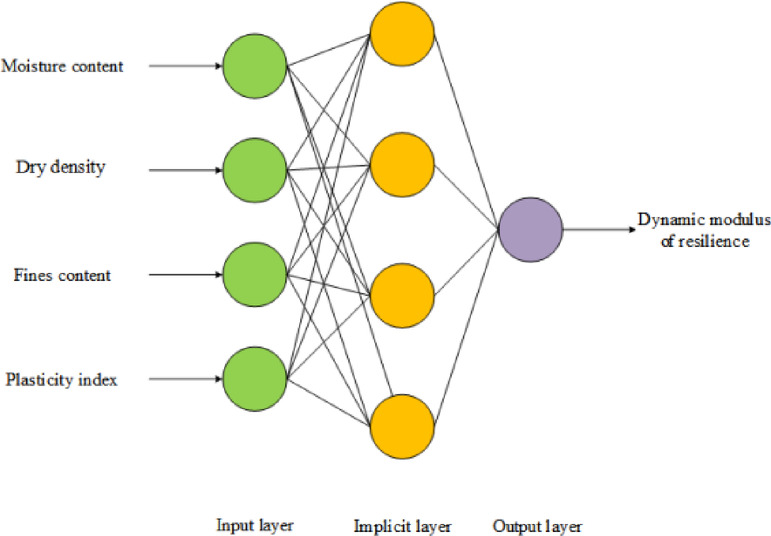
ANN structure diagram.

In view of the many advantages of the model, AASHT02002^11^ has recommended it as a model for predicting the dynamic modulus of subgrade soils by conducting studies on the performance of subgrade pavements. The physical significance of *k*_1_, *k*_2_, and *k*_3_ in the above formula is not clear; different soil modeling parameters need to be obtained through the test. However, in the practical highway construction projects, there is generally a lack of conditions to carry out a large number of dynamic resilient modulus tests. While the water content, compaction, fine grain content, and other physical property indicators are relatively easy to obtain through a number of relatively simple geotechnical tests. In order to be able to reasonably determine the dynamic resilient modulus of fine-grained soils within the range along this highway under the premise of simple geotechnical tests, this paper intends to construct the relationship equation between the model parameter *k*_*i*_ and the physical property index and finally import it into the model of Eq. (2) for prediction. Based on the dynamic resilient modulus tests that have been conducted, the moisture content *w* (%), dry density *ρ*_d_ (g/cm^3^), plasticity index *I*_p_ (%), and fine particle content *P*_0.075_ (%) are selected as the physical property indicators. Considering the number of test data, the backpropagation neural network (ANN) was used, and a genetic algorithm (GA) was incorporated to accelerate the parameter search process, so as to obtain the relational equations of Fine-grained soils along the alignment *k*_*i*_ with *w, K*, *I*_p_, and *P*_0.075_. The idea of ANN-GA modeling is to take sets of specimen data from Table 2 as the training set, consider the trained ANN as a predictive function, construct the objective function (i.e., the fitness function) by combining the measured value of the dynamic resilient modulus, and then use the GA to perform a globalized search for optimization. The modeling process is shown in Fig. 8:

The structure of the established backward feedback ANN is shown in Fig. 9, with four nodes in the input layer, which are water content, dry density, fines content, and plasticity index. It contains one hidden layer, and the output layer is the dynamic resilient modulus *M*_r_. Thus, the neural network structure follows a 4–4–1 topology. The hidden layer nodes employ the following Tangent-sigmoid activation function:

3 $$f(x) = \frac{2}{{1 + e^{ - 2x} }} - 1$$

**Fig. 10:**
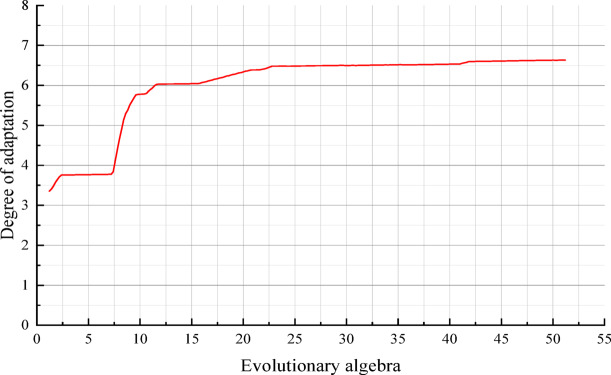
Fitness varies with evolutionary generation.

Subsequently, the objective function (Eq. 4) is established as the fitness function. Genetic Algorithm (GA) is applied for optimization, with the evolutionary generation set to 50 generations and the crossover probability and mutation probability set to 0.4 and 0.2, respectively. The change curve of fitness of the best individual when searching through the GA is shown in Fig. 10, and the increase of fitness shows the increasing accuracy of the prediction of the established relation.

4 $$F{ = }n{/}\left[ {\sum\limits_{i = 1}^{n} {\left( {\frac{{M_{pi} - M_{mi} }}{{M_{mi} }}} \right)}^{2} } \right]$$

where *M*_*pi*_—predicted value of dynamic resilient modulus; *M*_*mi*_—measured value of dynamic resilient modulus.

**Fig. 11:**
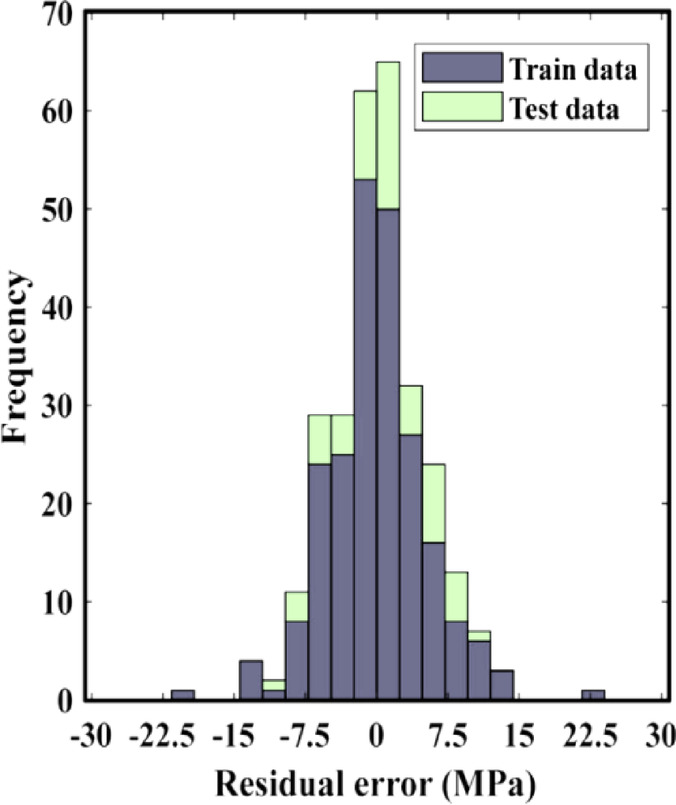
Model performance evaluation results.

To evaluate the performance of the obtained ANN-GA model, the residual error histograms of both the training and test sets are illustrated in Fig. 11. The residual errors are predominantly concentrated within the range of ± 7.5 MPa. It can be concluded that the developed model can accurately predict the resilient modulus of subgrade soils.

Equations (5) to (7) are the relationship equations between *k*_*i*_ and physical property indexes established using ANN-GA:

5 $$k_{{1}} = {1}.{1848}\rho_{{\text{d}}} - 0.0{349}w - 0.{1335}I_{{\text{p}}} - {1}.{15}0{9}P_{{0.0{75}}}$$

6 $$k_{{2}} = 0.00{25}\rho_{{\text{d}}} - 0.00{19}w + 0.00{24}I_{{\text{p}}} - 0.000{3}P_{{0.0{75}}}$$

7 $$k_{{3}} = 0.0{483}\rho_{{\text{d}}} - 0.0{225}w + 0.{5228}I_{{\text{p}}} - 0.{5166}P_{{0.0{75}}}$$

where *k*_1_ is the main parameter to control the size of *M*_r_. From the above equation, it can be seen that dry density *ρ*_d_ (g/cm^3^, positive correlation) and the fines content *P*_0.075_(%, negative correlation) have a greater degree of influence on it. *k*_2_ is the parameter to reflect the degree of influence of the body stress. The effects of *ρ*_d_*, w*(%)*,* and *I*_p_(%) are relatively even on *k*_2_, but the influence of *P*_0.075_ can be ignored. *k*_3_ is the parameter to reflect the degree of influence of octahedral shear stress *τ*_oct_, and *I*_p_ and *P*_0.075_ both have relatively large effects on it.

### Comparison with the results predicted by the canonical generalized formulas

**Fig. 12:**
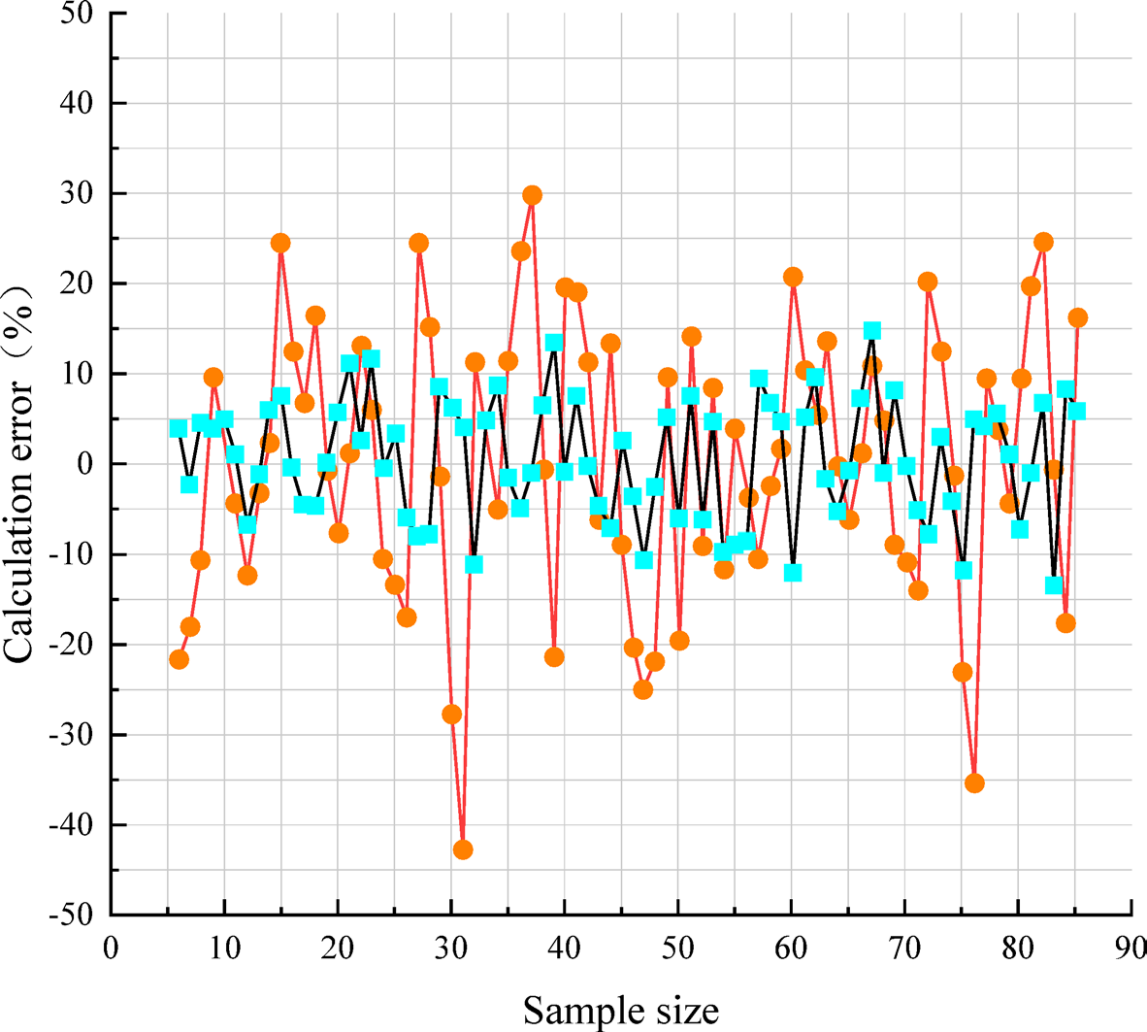
Comparison of calculation errors for a large number of specimens.

Substituting Eqs. (5) to (7) in Sect. 4.1 into Eq. (2), thus using the physical properties index of each group of specimens to predict their dynamic modulus of rebound, and then combined with the test-measured values, can calculate the prediction error, and then further compare the error with the prediction error of the standard general model^11^. The results are shown in Table 3 and Fig. 12.

**Table 3:** The prediction effect of different models for soil I

Test stress conditions		Dynamic modulus of resilience measured value (MPa)	Prediction error(%)	
Body stresses *θ* (kPa)	Octahedral shear stress *τ*_oct_ (kPa)		Specification of generic models	Model of this paper
75	14.14	94.78	− 12.55	2.36
120	35.36	88.86	16.89	− 4.35
120	14.14	97.96	17.34	6.12
165	35.36	95.77	− 11.34	− 10.35
165	14.14	102.02	17.76	7.56
190	25.93	100.81	− 14.34	3.89
240	49.50	82.30	− 11.80	− 2.32
210	14.14	105.85	− 7.11	4.35
235	25.93	105.30	6.47	3.98
Mean value of prediction error(%, absolute value)			12.84	5.03

As shown in Fig. 12, due to the geographical differences of the soil, the prediction error of the dynamic resilient modulus of the subgrade soil along the line of this paper using the standardized general model is large, with an average prediction error of 15.84%, and the prediction error of individual specimens is close to 45%. In contrast, using the model proposed in this study, the absolute average prediction error is 5.74%, and the maximum prediction error does not exceed 15%, so the prediction effect has been improved.

Due to the small variation of the equivalent stress level in the subgrade for different traffic load levels, the body stress *θ* = 70 kPa and *τ*_oct_ = 13 kPa are selected according to the requirements of the literature^12^. The model developed in this paper will be used in the construction of the subgrade of this highway, and it is possible to roughly determine the possible value of the resilient modulus of the subgrade that can be achieved by selecting a certain kind of soil for the filling so as to eliminate the fine-grained soils that have poor engineering properties. The model can also be used to analyze more accurately the stresses on the subgrade pavement under traffic loads. In summary, for specific highway subgrade construction projects, it is valuable to carry out a certain number of targeted dynamic resilient modulus tests on representative subgrade soils in advance and establish a prediction model based on physical properties indicators. The estimation model can be used to calculate the dynamic resilient modulus based on simple geotechnical tests such as the water content test and compaction test, which can further provide the basis for the selection of materials, the prediction of pavement fatigue life, and the design of subgrade pavement thickness.

## Conclusions

In this paper, the following conclusions are obtained through a series of dynamic resilient modulus tests and related prognostic modeling:

- For soil I, *M*_r_ decreased nonlinearly with increasing dynamic bias stress, ranging from 5.6 to 26.7% at the same circumferential pressure. *M*_r_ increased with increasing circumferential pressure, ranging from 8.7 to 36.8% at the same dynamic bias stress. *M*_r_ increased with increasing compaction, where the most obvious enhancement effect of increasing compaction on *M*_r_ was observed at optimal water content.
- With the increase of water content, *M*_r_ decreases nonlinearly. For soil No. I, *M*_r_ decreases by 2.1% on average for every 1% increase of water content. *M*_r_ is uniformly inversely proportional to *I*_p_, and the use of fine-grained soils with too high a plasticity index should be avoided as far as possible in the engineering for subgrade filling.
- Acoording to Spearman correlation coefficient values between various input features and the resilient modulus, it can be observed that, apart from the correlation coefficients for body stress and octahedral shear stress in MEPDG model, *M*_r_ has the strongest correlation with the moisture content *w* (%), dry density *ρ*_d_ (g/cm^3^), plasticity index *I*_p_ (%), and fine particle content *P*_0.075_ (%).
- Combined with ANN and GA algorithm, the relationship equation between *k*_*i*_ and water content *w* (%), dry density *ρ*_d_ (g/cm^3^), plasticity index *I*_p_ (%), and fines content *P*_0.075_ (%) can be established on the basis of a large number of dynamic resilient modulus test data, and then construct the dynamic resilient modulus estimation model based on the physical property index.
- Compared with the standardized general model, the average prediction error of the model established in this paper is 5.74%, and the maximum prediction error does not exceed 15%, which improves the prediction effect. Therefore, for specific highway subgrade construction projects, it is valuable to carry out targeted dynamic resilient modulus tests in advance to establish a prediction model based on physical property indexes. This model can further provide a basis for the selection of engineering materials, the prediction of pavement fatigue life, and the design of the thickness of the subgrade pavement.

## Data Availability

All data generated or analysed during this study are included in this published article.
